# KSHV G-protein coupled receptor vGPCR oncogenic signaling upregulation of Cyclooxygenase-2 expression mediates angiogenesis and tumorigenesis in Kaposi’s sarcoma

**DOI:** 10.1371/journal.ppat.1009006

**Published:** 2020-10-15

**Authors:** María Victoria Medina, Agata D´Agostino, Qi Ma, Pilar Eroles, Lucas Cavallin, Chiara Chiozzini, Daiana Sapochnik, Cora Cymeryng, Elizabeth Hyjek, Ethel Cesarman, Julian Naipauer, Enrique A. Mesri, Omar A. Coso

**Affiliations:** 1 Universidad de Buenos Aires, Facultad de Ciencias Exactas y Naturales, Departamento de Fisiología y Biología Molecular, Buenos Aires, Argentina; 2 CONICET-Universidad de Buenos Aires, Instituto de Fisiología, Biología Molecular y Neurociencias (IFIBYNE), Buenos Aires, Argentina; 3 UM-CFAR/ Sylvester CCC Argentina Consortium for Research and Training in Virally induced AIDS-Malignancies, University of Miami Miller School of Medicine, Miami, Florida, United States of America; 4 Tumor Biology Program, Sylvester Comprehensive Cancer Center and Miami Center for AIDS Research, Department of Microbiology and Immunology, University of Miami Miller School of Medicine, Miami, Florida, United States of America; 5 Laboratorio di Virologia, Istituto Superiore di Sanita, Rome, Italy; 6 Universidad de Buenos Aires, Consejo Nacional de Investigaciones Científicas y Técnicas (CONICET), Centro de Estudios Farmacológicos y Botánicos (CEFYBO), Facultad de Medicina. Buenos Aires, Argentina; 7 Universidad de Buenos Aires, Facultad de Medicina, Departamento de Bioquímica Humana, Buenos Aires, Argentina; 8 Department of Pathology, The University of Chicago, Chicago, Illinois, United States of America; 9 Department of Pathology, Weill Medical College of Cornell University, New York, New York, United States of America; University of Southern California, UNITED STATES

## Abstract

Kaposi’s sarcoma-associated herpesvirus (KSHV) vGPCR is a constitutively active G protein-coupled receptor that subverts proliferative and inflammatory signaling pathways to induce cell transformation in Kaposi’s sarcoma. Cyclooxygenase-2 (COX-2) is an inflammatory mediator that plays a key regulatory role in the activation of tumor angiogenesis. Using two different transformed mouse models and tumorigenic full KSHV genome-bearing cells, including KSHV-Bac16 based mutant system with a vGPCR deletion, we demostrate that vGPCR upregulates COX-2 expression and activity, signaling through selective MAPK cascades. We show that vGPCR expression triggers signaling pathways that upregulate COX-2 levels due to a dual effect upon both its gene promoter region and, in mature mRNA, the 3’UTR region that control mRNA stability. Both events are mediated by signaling through ERK1/2 MAPK pathway. Inhibition of COX-2 in vGPCR-transformed cells impairs vGPCR-driven angiogenesis and treatment with the COX-2-selective inhibitory drug Celecoxib produces a significant decrease in tumor growth, pointing to COX-2 activity as critical for vGPCR oncogenicity *in vivo* and indicating that COX-2-mediated angiogenesis could play a role in KS tumorigenesis. These results, along with the overexpression of COX-2 in KS lesions, define COX-2 as a potential target for the prevention and treatment of KSHV-oncogenesis.

## Author summary

Kaposi sarcoma (KS) is the most frequent AIDS-related cancer (AIDS-KS). The tumors originate in cells infected with a cancer-causing virus (KSHV). A gene encoded by the virus expresses a protein with oncogenic potential in the infected cells (vGPCR), which has the ability to promote cell transformation and angiogenesis driving KS tumorigenesis. Therefore, the identification of oncogenic intracellular signalling mechanisms triggered by vGPCR could be of therapeutic interest. Cyclooxygenase-2 (COX-2) is an inflammatory molecule involved in tumor angiogenesis that can be targeted by several FDA-approved non-steroidal anti-inflammatory drugs and specific inhibitors. Here, we demonstrate that vGPCR upregulates COX-2 activity and expression through upregulation of transcription and mRNA stability via an ERK1/2 dependent pathway. We also show that COX-2 activity is critical for vGPCR angiogenesis and oncogenesis using KSHV infection and mouse models. Consistent with a role in KS pathogenesis, we found that vGPCR upregulates COX-2 activity in endothelial cells, that it is essential for VEGF upregulation via vGPCR, and that it is expressed in KSHV infected cells of AIDS-KS lesions. These facts point to COX-2 as one of the molecular components of the vGPCR angiogenic switch in Kaposi Sarcoma and a potential target for chemoprevention and therapy.

## Introduction

Kaposi’s sarcoma (KS) is among the most common type of cancers associated with the Acquired Immunodeficiency Syndrome (AIDS, AIDS-KS) [1–4]. KS arises as multifocal lesions in the skin, lungs and gastrointestinal tract characterized by intense angiogenesis, spindle cell proliferation and erythrocyte extravasation [1–5]. Early KS lesions are treated with local and non-toxic therapies; however, advanced KS is treated with systemic chemotherapy, which is difficult to tolerate for AIDS patients [3,6,7]. Thus, the development of rational therapies based on KS pathogenesis is critical to fill this gap [3,6,8,9]. KS is caused by the KS herpesvirus (KSHV) or Human herpesvirus-8 (HHV-8), an oncogenic γ-2-herpesvirus, which carries several viral oncogenes responsible for the KS angiogenic phenotype [4,10–13]. KSHV encodes several open reading frames for proteins with the potential to regulate host-cell oncogenic signaling mechanisms [14]. ORF74 expression renders a constitutively active G-protein coupled receptor (vGPCR) homologous to angiogenic-chemokine receptors [15–17]. vGPCR has been identified as a major KSHV angiogenesis activating oncogene [18–20] that subverts host-cell proliferative and inflammatory signaling cascades leading to tumorigenicity and VEGF-mediated angiogenesis [18,21,22], and it has been shown to produce KS-like angioproliferative lesions in mice [20,23–25]. vGPCR immortalizes human endothelial cells by autocrine activation of the VEGF receptor [17] and upregulates PDGF expression via a Rac1-NOX-ROS oxidative stress axis [26]. All these facts identify vGPCR, and the molecular components of the pro-angiogenic signaling pathways that triggers, as targets for preventing and treating KS.

Cyclooxygenase-2 (COX-2) is an inflammatory mediator that plays a key regulatory role in the activation of tumor angiogenesis [27,28], and it is constitutively expressed in some human cancers [29,30], including KS lesions (skin tissue and lymph node) [31]. Moreover, Cyclooxygenase-2 (COX-2), is one of the host genes that is highly induced upon KSHV de novo infection of human microvascular endothelial cells (HMVEC-d) and human foreskin fibroblast (HFF) cells [31–35], and COX-2 silencing or chemical inhibition significantly reduces the proliferation and invasiveness of KSHV-infected endothelial cells [31, 32,34–38]. COX proteins catalyze the metabolism of arachidonic acid to produce prostaglandins as PGE-2 [29]. Accordingly, high levels of PGE2 secretion have been observed during KSHV primary infection [32,33] and PGE2 receptors have also been shown to be expressed in KS and PEL tissues [39]. Furthermore, COX-2 has been reported as a possible target of the drug nimesulide in PEL treatment [37].

Molecular mechanisms that regulate COX-2 promoter activation and mRNA stability by KSHV in relevant oncogenesis models are limited [31,35,40–43]. It has been shown that COX-2 gene expression can be down-regulated by EP2 and EP4 antagonist through its promoter or mRNA half-time regulation [39]. Moreover, *in vitro* angiogenic models and *in vivo* tumorigenic models are needed to further validate COX-2 and PGE2 inhibitors as novel KS therapeutic targets. Using two different transformed mouse-cell models and tumorigenic full KSHV genome-bearing cells, including KSHV-Bac16 based mutant system, we demostrate that vGPCR upregulates COX-2 expression and activity, signaling through selective MAPK cascades. We show that in addition to inducing COX-2 gene promoter activation, vGPCR can induce the stabilization of COX-2 mRNAs through ERK1/2 signaling pathways. We found that COX-2 plays a key role in vGPCR angiogenesis in murine skin and tumors indicating that COX-2 could be a link between vGPCR signaling and angiogenesis regulation. Moreover, using KS biopsies we show that COX-2 is overexpressed in KSHV-infected cells of KS lesions, defining COX-2 as a potential target for preventing and treating KSHV-oncogenesis.

## Results

### vGPCR regulates COX-2 activity and expression

**Fig 1:**
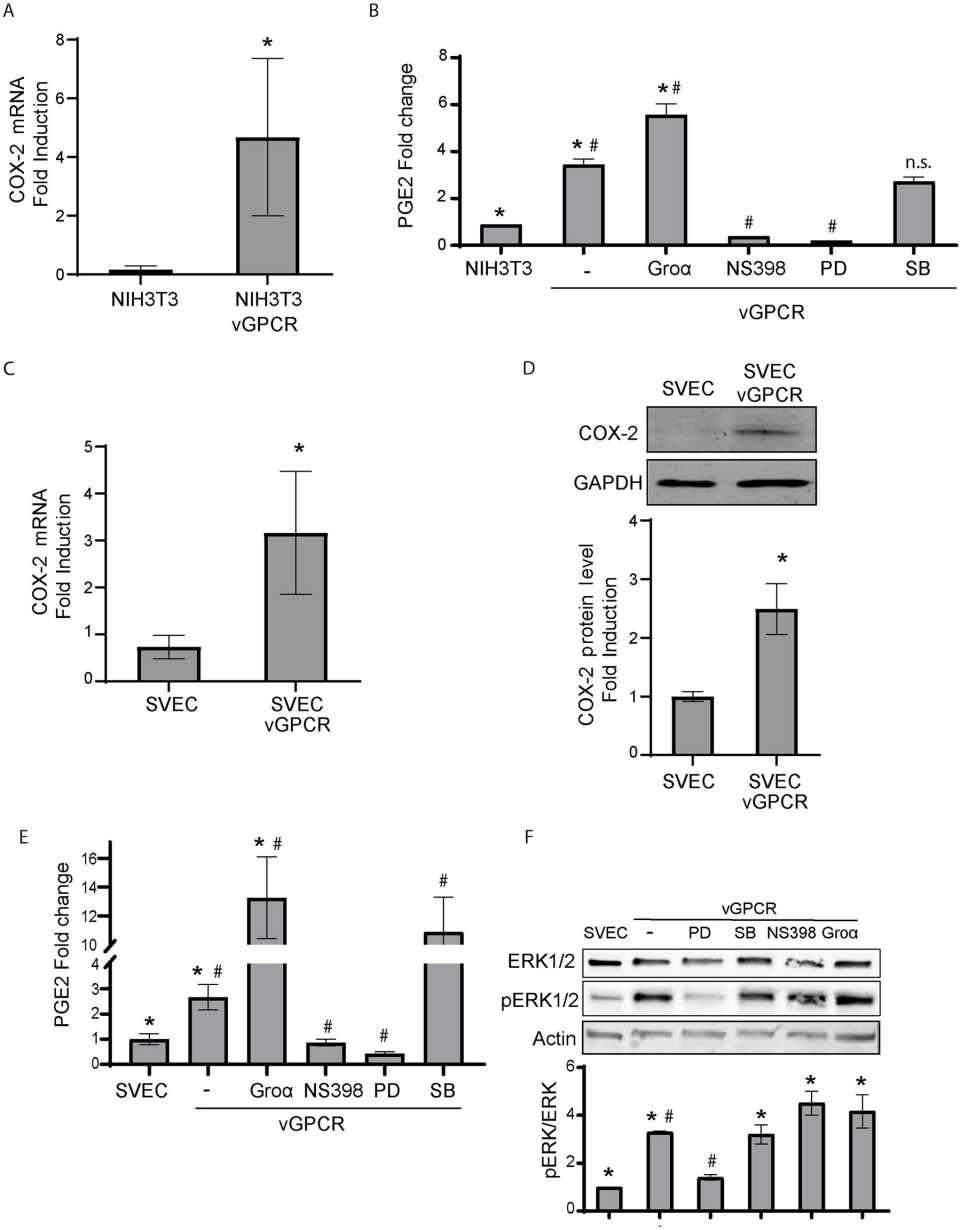
vGPCR oncogene expression increases COX-2 mRNA and protein expression levels as well as COX-2 activity. **A)** Fold-changes of COX-2 mRNA expression in transformed NIH3T3 (NIHT3T3-vGPCR) cells that stably express the vGPCR oncogene and control cells were assessed by RT-qPCR in triplicate and are presented as means ± SD. (*P <0.05). **B)** NIH3T3 cells were transfected with vGPCR expression vectors and were incubated ON in serum-free media. The vGPCR full agonist Gro-α (25nM), the COX-2 inhibitor NS398 (10uM), the ERK1/2 MAPK inhibitor PD98059 (20uM), or the p38 inhibitor SB220025 (10uM) were added to the cells as indicated. COX-2 activity was assessed measuring PGE2 production in the supernatants by an ELISA. Bars indicate mean PGE2 production of duplicate determinations ± SD. (*) Indicates significant differences between NIH3T3 control cells and the NIH3T3-vGPCR group of samples (*P*<0.05). (#) Indicates significant differences between sets of NIH3T3-vGPCR cells (*P*<0.05). **C)** Fold-changes of COX-2 mRNA expression in transformed SVEC (SVEC-vGPCR) cells that stably express the vGPCR oncogene and control cells were assessed by RT-qPCR in triplicate and are presented as means ± SD. (*P <0.05). **D)** COX-2 protein expression levels were determined by immunoblotting in SVEC cells that stably express the vGPCR oncogene. GAPDH was used as a loading control. COX-2 protein levels were measured in triplicate and are presented as means ± SD. (*P <0.05). **E)** SVEC cells were transfected with vGPCR expression vectors and were incubated ON in serum-free medium. The vGPCR full agonist Gro-α (25nM), the COX-2 inhibitor NS398 (10uM), the ERK1/2 MAPK inhibitor PD98059 (20uM), or the p38 inhibitor SB203580 (10uM) were added to the cells as indicated. COX-2 activity was assessed measuring PGE2 production in the cell supernatants by an ELISA. Bars indicate mean PGE2 production of duplicate determinations ± SD. (*) Indicates significant differences between samples from SVEC control cells and the SVEC-vGPCR group of samples (*P*<0.05). (#) Indicates significant differences between sets of SVEC-vGPCR cells (*P*<0.05). **F)** Total and phospho-ERK1/2 levels were determined by immunoblotting in SVEC cells transfected with vGPCR. The ERK1/2 MAPK inhibitor PD98059 (20uM), the p38 inhibitor SB203580 (10uM), the COX-2 inhibitor NS398 (10uM), or the vGPCR full agonist Gro-α (25nM) were added to the cells as indicated. Actin was used as loading control. pERK1/2 levels related to Total ERK1/2 levels were measured in triplicate and are presented as means ± SD. (*) Indicates significant differences between samples from SVEC control cells and the SVEC-vGPCR group of samples (*P*<0.05). (#) Indicates significant differences between sets of SVEC-vGPCR cells (*P*<0.05).

We and others have found that vGPCR induces angiogenesis and tumorigenesis in NIH3T3 cells and SV-40 Large T antigen immortalized mouse endothelial cells (SVECs) [18,20]. COX-2 is an important inflammatory mediator of tumor angiogenesis and an attractive chemoprevention target that could be inhibited by many Nonsteroidal Anti-Inflammatory Drugs (NSAIDS). We analyzed the expression of COX-2 in cells that express KSHV-encoded vGPCR. We observed a strong upregulation of COX-2 mRNA in vGPCR expressing cells respect to control cells (Fig 1A). To study whether vGPCR can upregulate COX-2 activity, we compared the ability of vGPCR-expressing cells versus control cells to produce prostaglandin E2 (PGE2), using PGE2 enzyme immunoassay. We found that PGE2 synthesis was four times higher in vGPCR-expressing cells than in NIH3T3 controls (Fig 1B). To determine whether vGPCR signaling was directly involved in increasing COX-2 activity, we used the vGPCR full agonist Gro-α and found that vGPCR stimulation by Gro-α enhanced PGE2 production in vGPCR-expressing cells. NS398, a specific COX-2 inhibitor, blocked vGPCR induced PGE2 synthesis, suggesting that vGPCR-induced PGE2 production is dependent on the expression of an active COX-2. Taken together, these results indicate that vGPCR signaling stimulates COX-2 activity and consequent PGE2 production in NIH3T3 cells.

We next studied the ability of vGPCR expression to upregulate COX-2 in SVECs, a mouse endothelial cell system that was previously used to characterize mechanisms of vGPCR induced angiogenesis and tumorigenesis in a relevant target cell type [20]. We analyzed SVEC cell lysates both by RT-qPCR and immunoblot using specific primers and a murine COX-2 monoclonal antibody. We found an increase in mRNA and protein levels of COX-2 in vGPCR-transformed cells respect to control cells (Fig 1C and 1D), which correlates with an increase in COX-2 activity (Fig 1E) as already shown for NIH3T3 cells.

Since vGPCR constitutive signaling activates members of MAP kinase family cascade [18,21,44], we analyzed whether vGPCR activates COX-2 via p38 or ERK1/2. We found that vGPCR-expressing cells treated with the ERK1/2 inhibitor PD98059 showed a marked decrease in the levels of COX-2 activity. In contrast, while the p38 inhibitor SB220025 did not have any significant effect in NIH3T3-vGPCR cells (Fig 1B) it rendered upregulation of PGE2 secretion in SVEC-vGPCR cells (Fig 1E). Both pieces of information support that p38 MAPK is not involved in the signaling pathway that leads to increased COX-2 activation. MAPK activation was confirmed by the determination of ERK1/2 phospho-protein levels. We found that ERK1/2 was potently and significantly activated by phosphorylation only in vGPCR-expressing cells (Fig 1F). The vGPCR-triggered activation of ERK1/2 was confirmed by using the MAPK pharmacological inhibitor to MAPKK MEK1, PD98059 which impairs ERK1/2 MAPK signaling and significantly reduced and almost completely abolished ERK1/2 activation in vGPCR expressing cells. On the other hand, NS398 (a well-known COX-2 inhibitor) and SB203580 (a well-known p38 MAPK inhibitor) showed no effect (Fig 1F). Gro-α treatment did not increase ERK1/2 phosphorylation levels further to what vGPCR already increases. Previous results with vGPCR expressing HUVECs [17] showed that in cells where basal levels of ERK1/2 due to constitutive signaling were undetectable, Gro-α superactivation led to ERK1/2 phosphorylation. Since in the case of SVECs vGPCR expression and constitutive signaling already stimulates ERK1/2 (Fig 1F lanes 1 and 2), it is likely that the apparent lack of Gro-α stimulation of ERK1/2 in SVEC cells is due to the maximal stimulation and saturation of the ERK1/2 signaling pathway already achieved by the over expression of vGPCR in this system. Taken together our data shows that vGPCR signaling upregulates COX-2 expression and activity via ERK1/2.

### vGPCR upregulates COX-2 transcription via ERK1/2

**Fig 2:**
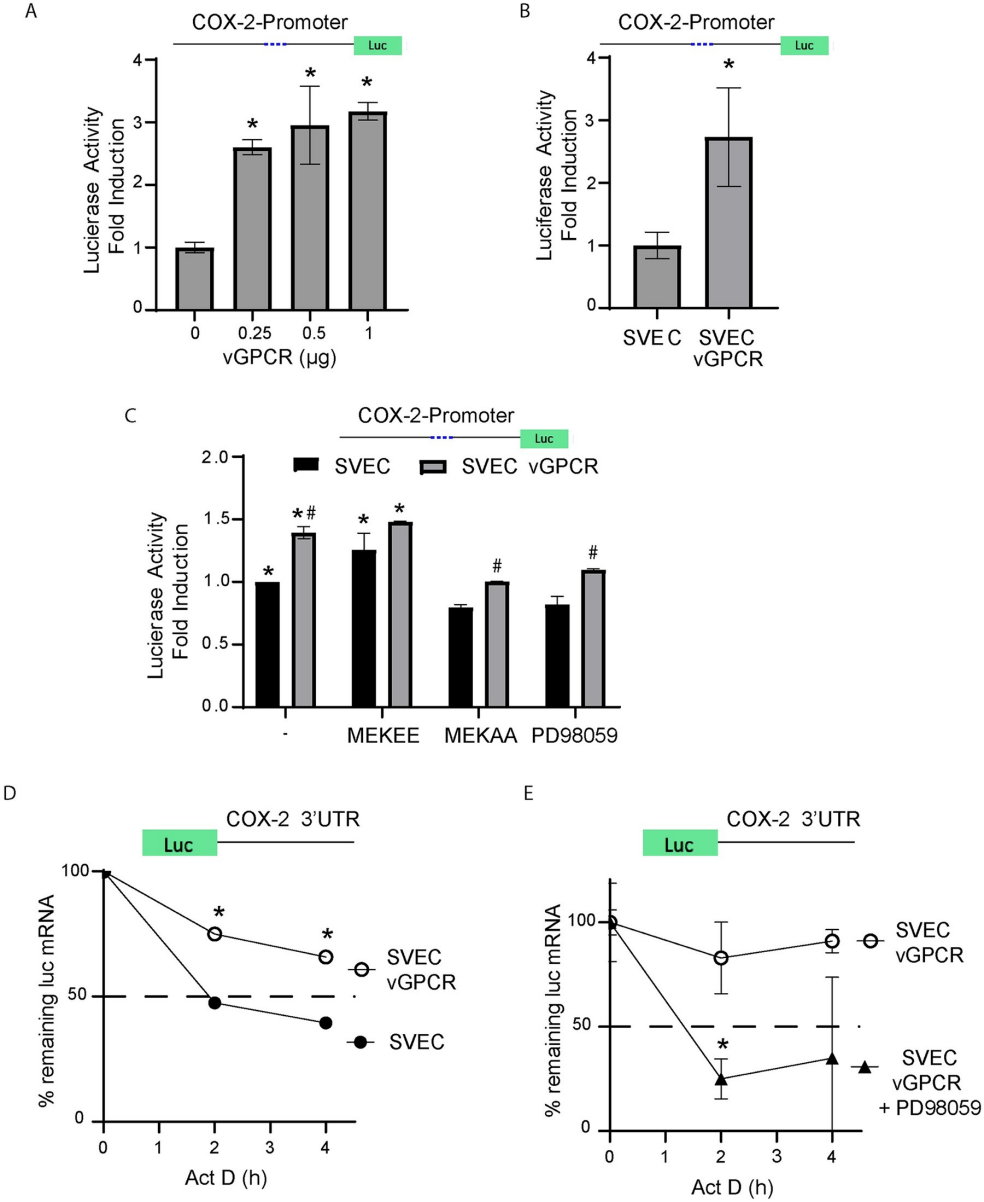
vGPCR signaling regulates COX-2 promoter activity and induces mRNA stability via ERK1/2. **A)** SVEC cells were transfected at increasing concentrations with a vGPCR expression vector and a luciferase reporter plasmid under the control of the COX-2 promoter. Luciferase activity expressed as fold induction relative to control cells that do not express vGPCR. Luciferase activity was measured in triplicate and is presented as means ± SD. (*P<0.05). **B)** Stably transfected SVEC-vGPCR cells and control cells were transfected with a luciferase reporter plasmid under the control of the COX-2 promoter. Luciferase activity was measured in triplicate and is presented as means ± SD. (*P<0.05) and expressed as fold induction relative to control cells. **C)** A reporter that expresses Luciferase under the control of a COX- 2 gene promoter region was co-transfected with a vGPCR expression vector and plasmids expressing constitutively active and dominant negative MAP kinase kinases (MEKEE and MEKAA respectively) or treated with the MEK/ERK1-2 inhibitor PD98059. Luciferase activity was tested and presented as fold induction relative to SVEC control cells. (*) Indicates significant differences relative to SVEC control untreated cells (*P*<0.05). (#) Indicates significant differences relative to SVEC-vGPCR untreated cells (*P*<0.05). **D)** mRNA stability assays were performed using a reporter plasmid containing the COX-2 3’UTR region cloned downstream of the luciferase ORF from SVEC or SVEC-vGPCR cells. Actinomycin D (5 *μ*g/*m*l) was added (t = 0) to arrest transcription, and mRNA levels of Luciferase mRNA were analyzed by qRT-PCR following a time course (4 hours). Luciferase mRNA was measured in triplicate and is presented as means ± SD. (*P <0.05). **E)** mRNA stability assays in SVEC-vGPCR cells transfected with the same reporter plasmid as in D) in the presence or absence of the MEK/ERK1-2 inhibitor PD98059 (20 uM). Actinomycin D (5 *μ*g/*m*l) was added (t = 0) to arrest transcription, and mRNA levels of Luciferase mRNA were analyzed by qRT-PCR following a time course (4 hours). Luciferase mRNA was measured in triplicate and is presented as means ± SD. (*P<0.05).

To evaluate if the vGPCR upregulation of COX-2 was due to transcriptional activation, we used a luciferase reporter under the control of an active portion of the COX-2 promoter [45]. Shelby et al. have provided evidence that KSHV vGPCR induces COX-2 transcription in primary vascular endothelial cells [41]. We found that both vGPCR transient and stable expression in SVECs upregulates COX-2 promoter activity (Fig 2A and 2B). Since ERK1/2 activation is known to regulate gene transcription, we analyzed by qRT-PCR whether vGPCR up-regulation of COX-2 mRNA expression was MAPK dependent. Pharmacological inhibition of the ERK1/2 signaling pathway was achieved by adding the specific inhibitor of the MAPKK MEK1 PD98059 (Fig 2C). Alternatively, as the use of pharmacological inhibitors may have potential off-target effects, we used a different method to validate these findings. Cells were transfected with constitutively active or dominant negative (kinase-dead) MEK1 mutants (MEKEE and MEKAA, respectively). While activation of the MEK1-ERK1/2 axis increased COX-2 promoter activity *per se*, both the pharmacological inhibitor and the dominant negative MEK1 mutant showed a reduction of vGPCR induced luciferase activity. Overall, results in Fig 2C support the involvement of the MEK1-ERK1/2 axis in signaling from vGPCR to the COX-2 promoter.

### vGPCR signaling induces COX-2 mRNA stability

The stability of the COX-2 messenger RNA (mRNA) transcript has been shown to be mediated by p38/MK2 dependent signaling acting on the ARE sequences in the 3′ UTR region of the COX-2 mRNA [46]. Working with a plasmid that expresses luciferase fused to the 3’-UTR of COX-2 mRNA, we showed that vGPCR expressing cells induce stabilization of mRNAs containing the COX-2 3’UTR (Fig 2D). This mRNA stabilization is ERK1/2 MAPK dependent as it was abolished in the vGPCR expressing cells upon treatment with the MEK inhibitor PD98059 (Fig 2E).

### Using full KSHV genome bearing cells to analyze COX-2 expression regulation by vGPCR

**Fig 3:**
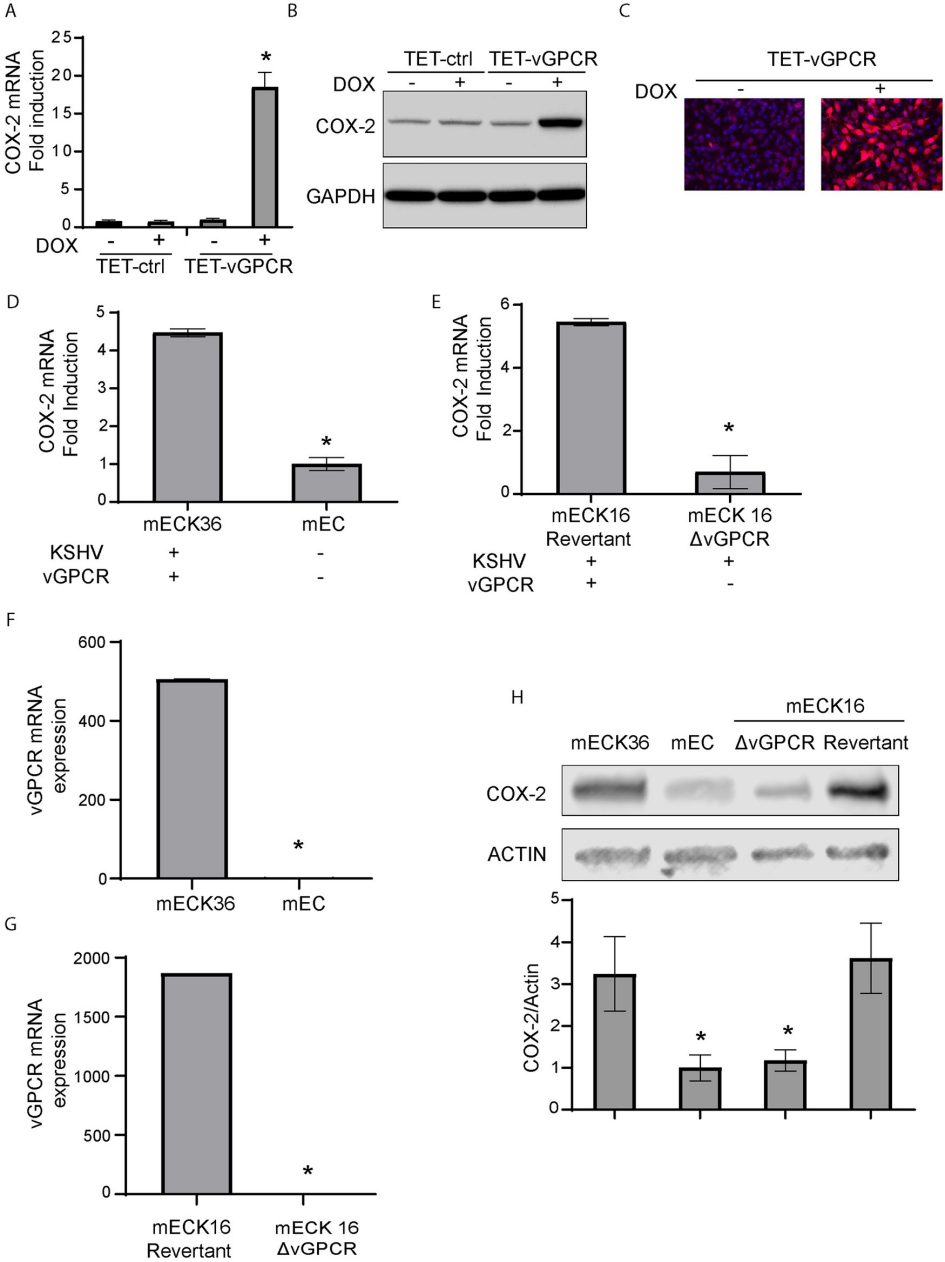
Use of full KSHV genome bearing cells to analyze COX-2 expression regulation by vGPCR. **A)** Fold-changes of COX-2 mRNA expression determined by RT-qPCR in Tetracycline-inducible vGPCR (TET-vGPCR) and control mECK36 cells stimulated with doxycycline for 24 hours. COX-2 mRNA was measured in triplicate and is presented as means ± SD. (*P<0.05). **B)** COX-2 protein expression levels were determined by immunoblotting in Tetracycline-inducible vGPCR (TET-vGPCR) and control mECK36 cells stimulated with doxycycline for 24 hours. GAPDH was used as a loading control. **C)** IFA for COX-2 (red) in Tetracycline-inducible vGPCR (TET-vGPCR) and control mECK36 cells stimulated with doxycycline for 24 hours. Cell nuclei were counterstained with DAPI (blue). **D)** Fold-changes of COX-2 mRNA expression determined by RT-qPCR in mECK36 and mEC cells (originated from the former and generated by selection of those that have lost the KSHVBAC36). COX-2 mRNA was measured in triplicate and is presented as means ± SD. (*P<0.05). **E)** Fold-changes of COX-2 mRNA expression determined by RT-qPCR in mECK16 derived Δ-vGPCR or revertant virus (see Materials and methods). COX-2 mRNA was measured in triplicate and is presented as means ± SD. (*P<0.05). **F)** vGPCR mRNA expression determined by RT-qPCR in mECK36 and mEC cells (KSHV-negative cells originated from the former and generated by selection of those that have lost the KSHVBAC36). The lowest CT value obtained in KSHV-negative mEC cell samples was assigned as the limit of detection for vGCPR expression. vGPCR mRNA was measured in triplicate and is presented as means ± SD. (*P<0.05). **G)** vGPCR mRNA expression determined by RT-qPCR in mECK16 derived Δ-vGPCR or revertant virus (see Materials and methods). The lowest CT value obtained in mECK16 Δ-vGPCR cell samples was assigned as the limit of detection for vGCPR expression. vGPCR mRNA was measured in triplicate and is presented as means ± SD. (*P<0.05). **H)** COX-2 protein expression levels were determined by immunoblotting in mECK36, mEC and mECK16 derived Δ-vGPCR or revertant virus (see Materials and methods). COX-2 protein levels were measured in triplicate and are presented as means ± SD. (*P<0.05).

KSHV harbors several open reading frames that encode proteins with the potential to regulate host-cell oncogenic signaling mechanisms. To study the regulation of COX-2 by vGPCR in the context of cells bearing the full KSHV genome we used tetracycline-inducible vGPCR (TET-vGPCR) overexpression in a mouse bone-marrow endothelial-lineage cells (mEC) transfected with the KSHVBac36 (a bacterial artificial chromosome that contained the whole KSHV genome), mECK36 cells [26]. Similar to the observation in vGPCR transformed cells, in the presence of KSHV, vGPCR overexpression leads to a potent COX-2 mRNA and protein upregulation as detected by qRT-PCR (Fig 3A), Western blots (Fig 3B) and IFA (Fig 3C).

**Fig 4:**
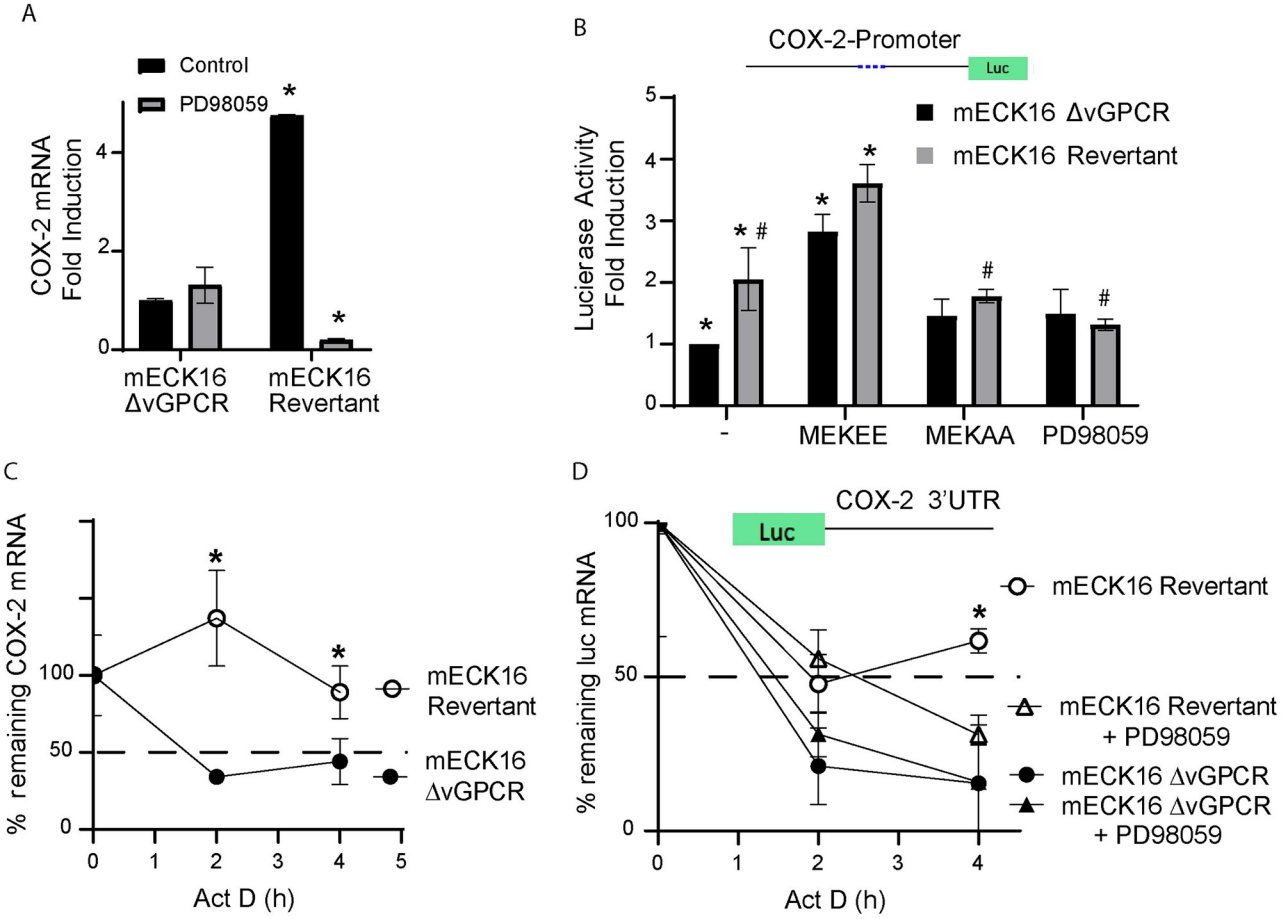
vGPCR regulates COX-2 promoter activity and mRNA stability via ERK1/2 in full KSHV genome bearing cells. **A)** Fold-changes in mRNA expression determined by RT-qPCR in mECK16 derived cells (Δ-vGPCR or revertant virus) after treatment with ERK1/2 MAPK inhibitor PD98059 (20uM). COX-2 mRNA was measured in triplicate and is presented as means ± SD. (*P<0.05). **B)** mECK16 derived Δ-vGPCR and revertant cells were transfected with a luciferase reporter plasmid under the control of the COX-2 promoter (as in Fig 2A). Luciferase activity is expressed as fold induction relative to control cells. Cells were co-transfected with plasmids expressing constitutively active and dominant negative MAP kinase kinases (MEKEE and MEKAA respectively) or treated with the MEK/ERK1-2 inhibitor PD98059. (*) Indicates significant differences from mECK16 Δ-vGPCR untreated cells (*P*<0.05). (#) Indicates significant differences between mECK16 revertant untreated cells (*P*<0.05). **C)** mRNA stability assay in mECK16 derived (Δ-vGPCR and revertant) cells. Actinomycin D (5 μg/ml) was added (t = 0) to arrest transcription, and mRNA levels of COX-2 were analyzed by qRT-PCR following a time course (4 hours). COX-2 mRNA was measured in triplicate and is presented as means ± SD. (*P<0.05). **D)** mRNA stability assay in mECK16 derived (Δ-vGPCR and revertant) cells transfected with a reporter plasmid containing the COX-2 3’UTR region cloned downstream of the luciferase ORF in the presence or absence of the MEK/ERK1-2 inhibitor PD98059 (20 uM). Actinomycin D (5 μg/ml) was added (t = 0) to arrest transcription, and mRNA levels of Luciferase were analyzed by qRT-PCR following a time course (4 hours). Luciferase mRNA was measured in triplicate and is presented as means ± SD. (*P<0.05).

To determine the specific contribution of vGPCR signaling to the expression of COX-2 in the context of KSHV, we used the Bac16 based mutant system. We used the procedure described in Ashlock et al. to “swap” Bac36 for the Bac16-delta vGPCR mutant or its revertant in mECK36 cells that have lost the Bac36 episome by lack of antibiotic selection (KSHV-negative cells, mEC) (See Materials and methods) [47], to generate the cell lines mECK16-ΔvGPCR and mECK16-revertant, respectively. Fig 3D and 3H show a sharp drop in COX-2 mRNA and protein expression when mECK36 cells lose the KSHV episome indicating that, as shown in other infection systems, KSHV induces COX-2 upregulation. We found a similar drop in COX-2 expression between Bac16-revertant bearing cells and the vGPCR deletion mutant (Fig 3E and 3H). Since LANA and vFLIP has been shown to activate COX-2 [35] and in order to rule out effects in COX-2 regulation due to downregulation of these viral genes caused by vGPCR deletion in the Bac16 mutant, we assessed their expression levels by qRT-PCR. Interestingly, vGPCR deletion mutant showed more expression of LANA and similar expression levels of vFLIP than mECK16 revertant cells (S1 Fig). Taken together these results reinforce the idea that the effects on COX-2 downregulation in the Bac16 mutant are due to vGPCR deletion. Importantly, in contrast to mEC and Bac16Δ-vGPCR that showed no vGPCR expression, mECK36 cells and Bac16-revertant showed vGPCR expression as detected by qRT-PCR (Fig 3F and 3G). This data strongly suggests that most of the COX-2 upregulation in mECK36 and KSHVBac16 bearing cells is due to the presence of the vGPCR oncogene. Inhibition of MAPK signaling in this cellular setting by the addition of the MEK inhibitor PD98059 is consistent with data already shown in this study (Fig 2C), indicating that COX-2 activation by vGPCR is dependent on ERK1/2 signaling, as we found a downregulation of COX-2 expression in Bac16-revertant bearing cells treated with the MEK inhibitor (Fig 4A). Finally, we tested COX-2 promoter activity and mRNA stability in the KSHVBac16 cells expressing or not vGPCR, and we found that in the context of KSHV, vGPCR is a major contributor to COX-2 expression by triggering signaling that induces COX-2 promoter activity and mRNA stability via ERK1/2 MAPK (Fig 4B and 4C). Working with a plasmid that expresses luciferase fused to the 3’-UTR of COX-2 mRNA, we showed that vGPCR expressing cells induce stabilization of mRNAs containing the COX-2 3’UTR (Fig 4D). This vGPCR-driven mRNA stabilization is ERK1/2 MAPK dependent as it was abolished in the vGPCR expressing cells and not in vGPCR mutant cells upon treatment with the MEK inhibitor PD98059 (Fig 4D).

### COX-2 regulates vGPCR angiogenicity

**Fig 5:**
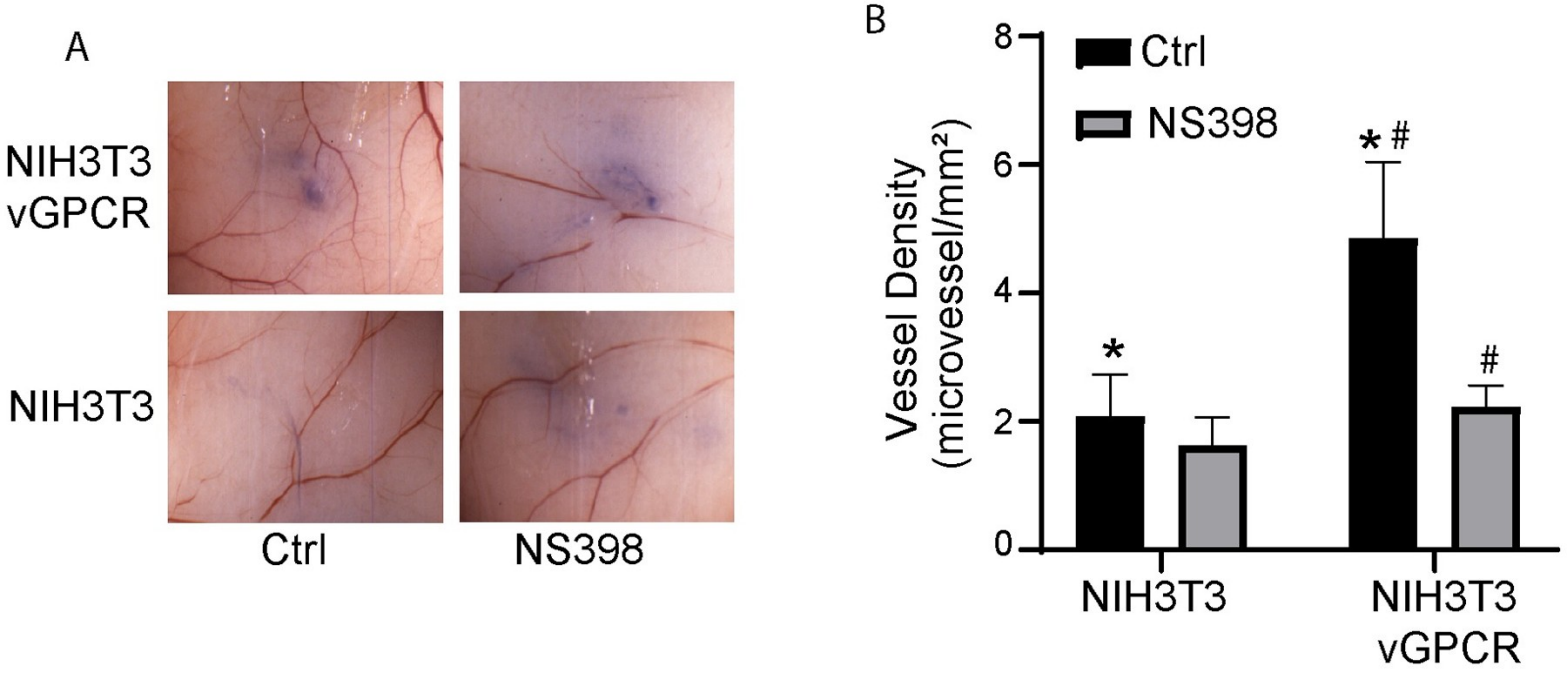
COX-2 regulates vGPCR angiogenicity. **A)** Cells expressing vGPCR and corresponding controls were treated or not with NS398 (10 mM) and inoculated I.D. into both flanks of nude mice (*n* = 5). Mice were sacrificed after 5 days and the area of inoculation was photographed under a dissection microscope. **B)** Neovessel formation determined by morphometric analysis. The bar graphs show the mean microvessel density (vessels/mm^2^) for NS398 pre-treated (grey bars) or untreated (black bars) cells +/-SD. Total n (both flanks) = 10. (*) Indicates significant differences between groups injected with vGPCR-transformed cells and NIH3T3 control cells (*P*<0.05). (#) Indicates significant differences between groups injected with NS398-treated and non-treated vGPCR-transformed cells (*P*<0.01).

COX-2 expression has been shown to regulate the angiogenicity of tumor cells [27]. Since vGPCR is an angiogenesis activator [18] and vGPCR activates COX-2 in NIH3T3 and SVEC cells (Fig 1), we sought to determine whether COX-2 activity modulates the angiogenesis induced by vGPCR in transformed cells using an intradermal angiogenesis assay [48]. We inoculated one group of nude mice with vGPCR-transformed NIH3T3 cells and another with vGPCR-transformed NIH3T3 cells pre-treated with NS398, a COX-2 inhibitor. Trypan blue exclusion was used to ensure that the same number of viable cells were injected in all the animals and to mark the site of injection. In parallel, cells treated or not with NS398 were maintained in culture for the duration of the animal experiment to rule out effects due to decreased cell viability. Mice inoculated with vGPCR-transformed NIH3T3 cells induced neo-vasculature intricacy and more vessel density than NIH3T3 cells inoculated mice (Fig 5A) which only presented straight mature vessels at the inoculation in their skin. Like these NIH3T3 controls, NS398 pretreated vGPCR-transformed cells did not induce microvessel proliferation at the site of inoculation (Fig 5A). Morphometric quantification and statistical analysis of the angiogenic response showed a significant increase of more than two-fold in the density of vessels determined on the skin of the group of mice injected with vGPCR-transformed cells (Fig 5B). On the other hand, vessel density in the group inoculated with vGPCR-transformed cells pre-treated with NS398 did not show significant differences compared to group injected with NIH3T3 control cells (Fig 5B). This indicates that treatment of the cells with NS398 before inoculation abolished the angiogenic response induced by vGPCR expression, suggesting that a COX-2 dependent pathway has a major contribution to *in vivo* angiogenesis mediated by vGPCR signaling in NIH3T3 cells.

### COX-2 mediates vGPCR tumorigenicity

**Fig 6:**
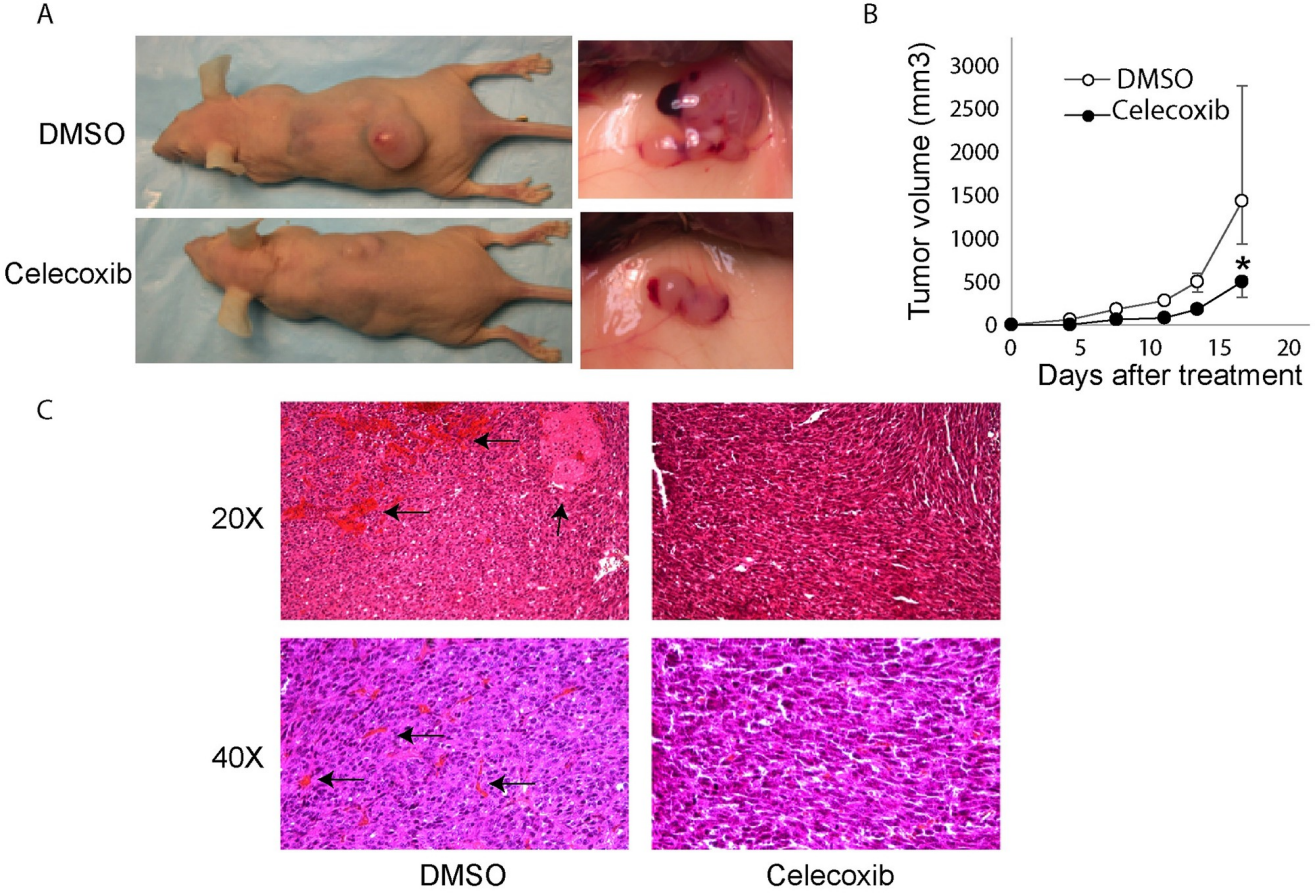
vGPCR-transformed cells tumorigenicity is inhibited by Celecoxib treatment. **A)** Mice (n = 7) were injected S.C. with vGPCR-NIH3T3 cells and treated with the COX-2 inhibitor Celecoxib I.P. or vehicle (DMSO) three times/week. On the left side, images of mice treated (lower) or not (upper) with Celecoxib at day 15 of treatment. Tumor from non-treated (upper) or treated with Celecoxib (lower) mice are shown in detail on the right. **B)** The plot shows the growth of tumor volume during the time of treatment (mean +/-range). Higher deviation in the last day for untreated animals reflect the presence of animals with very large tumors by that day observed in all the experiments. Mice were treated with Celecoxib (black circles) or vehicle (white circles). Tumor size was significantly lower in Celecoxib treated samples at all points of the time course (*P<0.05). **C)** Histological examination of the tumors. Sections of tumors coming from mice, treated with Celecoxib or not (*DMSO*) as a control, were stained with Hematoxylin-Eosin. Pictures were taken at 20x or 40x magnification. Black arrows in left panels indicates large areas of hemorrhage and necrosis.

To analyze the contribution of COX-2 mediated angiogenesis to vGPCR-induced tumorigenesis [18], we investigated the effect of treatment with the COX-2 selective inhibitory drug Celecoxib on the growth of vGPCR-transformed NIH3T3 tumors [49]. To confirm that NIH3T3-vGPCR induced-tumors expressed COX-2, we performed COX-2 inmunostaining and found that these tumors showed a high COX-2 expression (S2 Fig) indicating that COX-2 is expressed in vGPCR induced tumors. We subcutaneously (S.C.) inoculated nude mice with vGPCR-transformed NIH3T3 cells and treated one group with Celecoxib intraperitoneally (I.P.) three times a week and another group with the vehicle (DMSO) as control. We found that Celecoxib produced consistent retardation in the occurrence of tumors and a significant decrease in tumor growth (Fig 6A and 6B). Histological analysis revealed that the untreated group presented large areas of hemorrhage and necrosis, attributable to the large size of the tumor and outgrowth of the vascular supply (Fig 6C, left panels black arrows). The group treated with Celecoxib did not show necrosis in the tumor and showed only marginal hemorrhage (Fig 6C, right panels). These results indicate that COX-2 contributes to the growth of tumors induced by vGPCR and that treatment of mice with Celecoxib was effective in reducing tumor growth.

### COX-2 modulates tumor angiogenesis by regulating VEGF production in tumor cells

**Fig 7:**
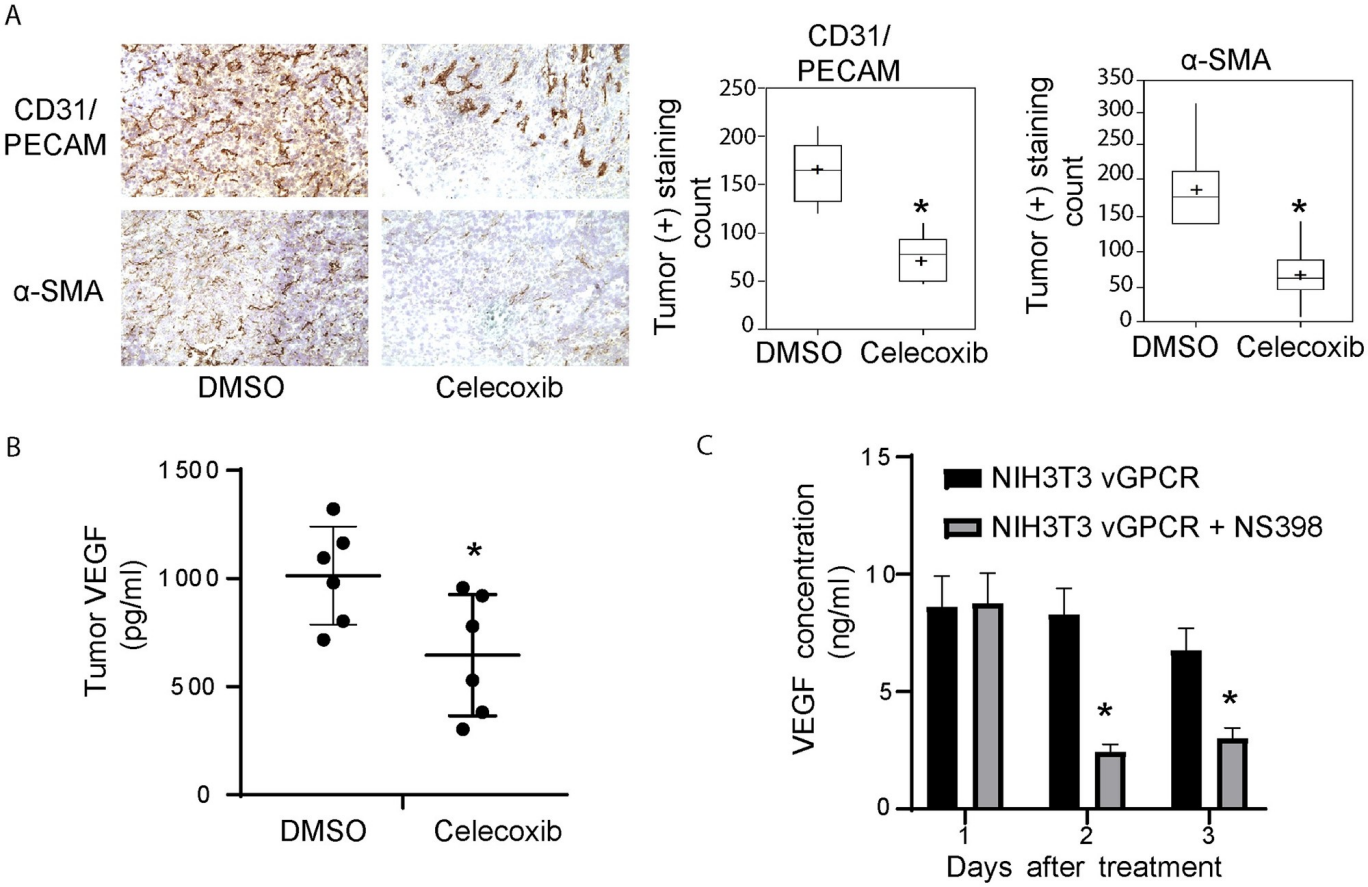
Celecoxib treatment inhibits vGPCR tumor angiogenesis and VEGF production in the tumor and transformed cells. **A)** Left panels: Immunoperoxidase staining for CD31/PECAM or SMA was performed on tumor sections. CD31/PECAM and SMA staining images of Celecoxib treated or untreated animals are shown. Right panel: Data of staining intensity levels is represented on box-plots showing the results for the morphometric quantification of CD31/PECAM (left) (*P<0.001) and αSMA (right) (*P<0.01) staining (*n* = 7). **B)** Tumor samples for mice tested for tumorigenesis were homogenized and centrifuged. VEGF production was measured in the supernatants by ELISA in Celecoxib treated mice or DMSO controls. The plot shows the mean of each group and the value of individual determinations (black circles) (*P<0.001) (*n* = 7). **C)** vGPCR-transformed NIH3T3 cells were cultured for the indicated time and treated (grey bars) or not (black bars) with NS398 (10uM). VEGF production was measured in the supernatants by ELISA. (*P<0.05).

To evaluate whether the anti-tumor effects caused by COX-2 inhibition were related to tumor angiogenesis inhibition, we quantified tumor vascularization by immunostaining. We used CD31/PECAM, a pan-endothelial marker, to stain tumor vessels as well as α-Smooth Muscle Actin (α-SMA) to stain pericyte-containing mature vessels [50]. Celecoxib treatment led to a dramatic reduction in both total (CD31+) and α-SMA+ vessels in NIH3T3-vGPCR tumors (Fig 7A, left panel), indicating that COX-2 inhibition greatly compromised vGPCR tumor angiogenicity. Statistical analysis of the data from immunostaining quantification (Fig 7A, right panel), indicate a significant decrease in CD31+ total vessels (163 +/-29 vs 73 +/-21) and SMA+ mature vessels (185 +/-63 vs 64 +/-40) in the tumors from animals treated with Celecoxib. These results show that Celecoxib treatment of mice reduced the total number of vessels in tumors and inhibited their maturation, indicating that a COX-2 dependent pathway contributes to vGPCR tumor angiogenicity.

Since our results *in vivo* indicated that COX-2 affects vGPCR angiogenicity (Fig 5) and tumorigenicity (Fig 6) and VEGF secretion is essential for tumor angiogenesis, we tested modulation of VEGF expression by COX-2 inhibitors in the tumors. We compared the levels of VEGF extracted from tumors of Celecoxib treated and untreated animals. Tumors from animals treated with Celecoxib produced significantly lower levels of VEGF than tumors from untreated animals (Fig 7B). Since COX-2 was shown to be able to regulate VEGF expression in transformed cells [27], we tested in cultured NIH3T3-vGPCR cells whether inhibition of COX-2 could affect VEGF secretion. We found that COX-2 inhibitors caused a dramatic reduction in VEGF secretion of NIH3T3-vGPCR without affecting cell growth (Fig 7C). Taken together our results suggest that COX-2 inhibition blocks tumor angiogenesis at least partly by inhibiting VEGF secretion by vGPCR expressing cells.

### COX-2 is overexpressed in KSHV-infected spindle cells in Kaposi’s sarcoma

**Fig 8:**
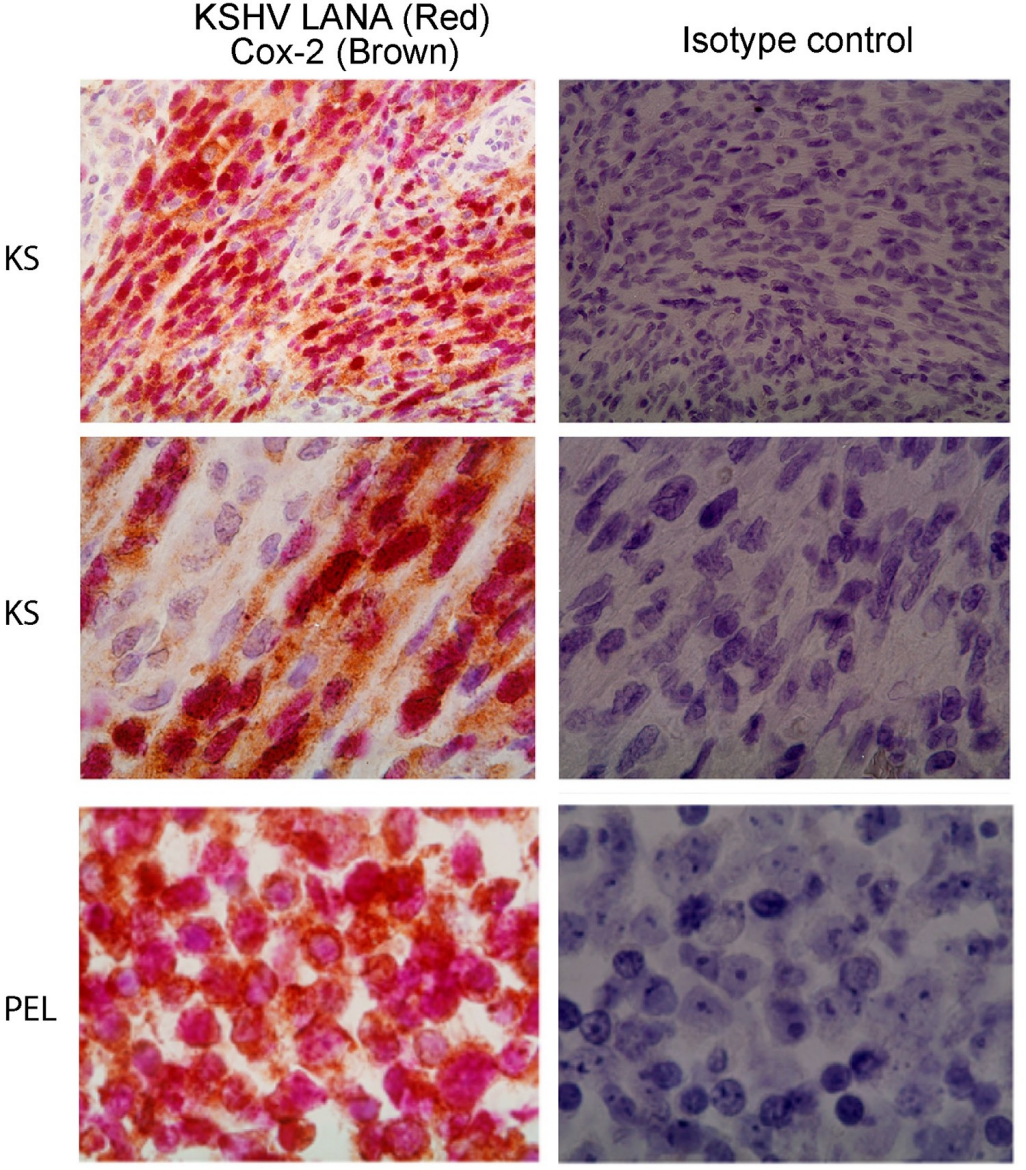
COX-2 is expressed in KSHV infected spindle cells of Kaposi’s sarcoma lesions. Sections from KS biopsies (two top panels, same patient at different magnification) or PEL (bottom panels) were incubated with COX-2 and KSHV LANA antibodies (left panels) or Isotype control (right panels). COX-2 (cytoplasmic) bound antibodies were developed with DAB (BROWN colored). KSHV LANA (nuclear) bound antibodies were developed with BP Red substrate (RED colored).

The activation of COX-2 by KSHV infection [31–33,35,51], and the involvement in vGPCR angiogenesis and tumorigenesis, as shown in this study, suggest that COX-2 could be a therapeutic target in KS. Primary to the definition of COX-2 as a KSHV-related therapeutic target is determining whether it is expressed in the majority of the KSHV infected spindle cells (LANA-positive) of KS lesions. To evaluate this possibility, we employed double-label immunohistochemistry for COX-2 and the KSHV latent nuclear antigen LANA in KS lesions. The PEL line BC-3 was used as a positive control for KSHV infected cells. We found that in all the KS lesions analyzed (5 out of 5) cytoplasmic staining of COX-2 (brown) was stronger in the spindle cells expressing LANA (red) than in either spindle cells that were LANA-negative or surrounding tissue (Fig 8, representative sample). These results indicate an association between COX-2 over-expression and KSHV infection in spindle cells of the human KS lesions.

## Discussion

Pathogenesis-based target identification has dramatically changed the way new therapies are developed. In the case of KS, the discovery of KSHV and identification of vGPCR as a major angiogenic oncogene provided a molecular signaling-cascade target for KSHV and Kaposi’s sarcoma. Using this insight, we have identified molecular signaling components of the cascades that are triggered by expression of the virus-encoded oncogene vGPCR and mediate its tumorigenic capabilities [17,18,26]. Results presented hereby support the idea of COX-2 as a mediator of vGCPR triggered cell-transformation and neovascularization that might constitute a suitable surrogate target for vGPCR angiogenesis and KSHV tumorigenesis.

The rationale for defining COX-2 as an emerging therapeutic and chemoprevention target for various cancers [52–54] includes experimental evidence for a role in carcinogenesis or angiogenesis using animal or cell models, together with evidence on the overexpression of COX-2 in tumor malignant cells [27,28,54]. Here we demonstrate that vGPCR induces COX-2 transcription and mRNA stabilization with the concomitant increase in COX-2 activity via ERK1/2 signaling pathway, and we show that COX-2 activity is critical for vGPCR pathogenicity *in vivo*. Moreover, we were interested in the role of COX-2 in vGPCR angiogenicity and oncogenicity as this inflammatory mediator can be targeted by low-toxicity NSAIDs in KSHV infected cells [31,35,37,38], which makes it an interesting surrogate target for chemoprevention of KS. We used the NSAID NS398 for our in vitro experiments because this is a very specific drug for COX-2. In contrast, we used the NSAID Celecoxib in our xenograft model because this is an FDA-approved drug that can be potentially repurposed for its use in clinical treatment of AIDS-KS.

We found that inhibition of COX-2 in cells with the NSAID NS398 impairs vGPCR-driven angiogenesis, in this intradermal angiogenic assay the cells are treated with the inhibitors and not the animals. Thus, this system is useful to evaluate the effects of the inhibitors in the angiogenic potential of the transformed cells by vGPCR without inhibiting the host cells (including endothelial cells). Moreover, this model mimics the physiologic conditions in which vGPCR would turn on an angiogenic response in the dermis, where KSHV-infected spindle cells are found. Treatment with the NSAID COX-2-selective inhibitory drug Celecoxib produced a significant decrease in tumor growth. Tumors from animals treated with Celecoxib showed a significant decrease in total and mature vasculature that correlated with a decrease in tumor cell VEGF production. We therefore conclude that vGPCR regulates angiogenicity and tumorigenicity via COX-2 activation. Consistent with a role in KS pathogenesis and with previous works showing COX-2 expression in KS lesions [31,55], we found that COX-2 is overexpressed in KSHV-infected (LANA-positive) spindle cells of human KS lesions. These facts point out COX-2 as a critical molecular component of the vGPCR transformation and angiogenic switch and as a potential target for KS chemoprevention and therapy.

In the context of vGPCR-transformed cells and full KSHV-genome bearing cells, we showed that a mitogenic pathway triggered by the vGPCR oncogene activates expression of COX-2 both at the gene promoter level and by increasing mRNA stability, with the involvement of MAPK signaling component (ERK1/2) in both cases. The regulation of gene expression by pathways acting both at the gene promoter level and by mRNA stabilization is an emerging concept that we have already observed studying c-Fos gene expression regulation [56]. Interestingly, KSHV proteins were shown to induce mRNA stability of host genes [57], including vGPCR-induced stabilization of COX-2 mRNA in non-transformed endothelial cells via p38/MK2 [40]. We showed that in the context of vGPCR-induced transformation and transformed full KSHV genome bearing cells, COX-2 transcription and mRNA stability are regulated mostly through vGPCR-triggered ERK1/2 signaling pathways. Transcription factors are the targets of MAPK signaling that activated in turn and induce gene promoter activation. Similarly, proteins named collectively as AUBPs bind to 3-UTR ARE regions of mRNAs, are MAPK targets and constitute regulatory determinants in the context of the control of mRNA stability. Identification of both the specific transcription factors and AUBPs involved in the regulation of COX-2 expression by vGPCR is the focus of further explorations.

Our results show that COX-2 plays a key role in vGPCR angiogenesis in a murine-skin assay and vGPCR induced tumors, indicating that COX-2 could be a link between vGPCR receptor signaling and angiogenesis regulation. COX-2 regulates angiogenesis in endothelial cells and inflammatory cells [58,59]. We provide evidence supporting that COX-2 inhibitors target COX-2-mediated vGPCR angiogenicity. As shown in Fig 5 pre-treatment with the COX-2 inhibitor NS398 repressed angiogenesis *in vivo* of vGPCR-transformed cells, indicating a direct role of COX-2 in the angiogenicity of the vGPCR-expressing cells. The contribution of COX-2 mediated angiogenesis to vGPCR tumorigenicity and angiogenicity is indicated by the impairment of tumor growth in Celecoxib treated animals that show significant decrease in tumor neo-vessel formation and maturation, correlated with intratumoral VEGF levels. Even though it is possible that Celecoxib could inhibit COX-2 involvement by inhibition of targets other than COX-2, we found that COX-2 inhibition impairs VEGF secretion in vGPCR-transformed cells (Fig 7) and we also demonstrated the decrease in tumoral VEGF in animals treated by Celecoxib. Taken together, our results provide evidence that supports that Celecoxib is targeting a COX-2 mediated pathway of VEGF production in vGPCR expressing tumor cells leading to angiogenesis inhibition.

Our data suggest that COX-2 could play a role in KSHV pathobiology and KS progression and is consistent with findings showing that COX-2 may play a role in MHV-68 replication [51] and is induced by de novo KSHV infection [31–33,35]. We found that COX-2 is expressed in the majority of latently-infected (LANA-positive) spindle cells of human KS lesions, as determined by the consistency of COX-2 cytoplasmic staining in spindle-cells displaying nuclear LANA staining (Fig 8). As generally only a small proportion of KS-spindle cells express the early lytic gene vGPCR; it follows that COX-2 should also be upregulated in latently infected vGPCR-negative cells, as shown in latently infected PEL cells where there is no vGPCR expression [38]. Within these vGPCR-negative cells, COX-2 upregulation could be caused by KSHV latent genes able to upregulate COX-2 such as LANA, vFLIP and K15 [35,42], by KSHV-dependent Nrf2 upregulation [60], or by paracrine induction of COX-2 growth factors as occurs in similar models where vGPCR expressing cells lead to secreted mediators such as PGE2 [61], VEGF [17] or PDGF [26]. These paracrine mechanisms could be prominent during the initial phases of KSHV tumorigenesis acting in a paracrine manner from either lytically or abortive-lytically KSHV infected cells [62], observed in mECK36 tumors [63] and reported for a portion of KS lesions [64]. Regardless of the precise mechanism of COX-2 expression in latently and lytically infected cells in KS lesions, our results confirm previous findings [31,55] regarding its expression in human KS lesions. This results, together with the expression of PGE2 receptors in KS [39], and the present identification of COX-2 activation as a key vGPCR oncogenic signaling component, further support the proposed role for COX-2 [34,37–39] as a therapeutic target in Kaposi’s Sarcoma.

Current frontline AIDS-KS therapy includes ART, which in advanced patients or ART-resistant individuals may additionally require systemic cytotoxic chemotherapy with liposomal anthracyclins [3,65]. Yet, it is estimated that more than half of these patients will not be cured [66] so new and less toxic treatment modalities are needed. Among the most promising targeted therapies, Rapamycin, an mTOR inhibitor that targets the KS paracrine oncogenesis axis and the multi-kinase inhibitor Imatinib which targets PDGFRA, c-kit and c-abl showed some responses in transplant [67] and AIDS-KS [68] respectively. Recently, immunomodulatory drugs as lenalidomide and pomalidomide [69] and some checkpoint inhibitors have shown prowess in AIDS-KS treatment [3]. The identification of KSHV oncogenes, and the host-cell signaling cascades dysregulated by them, unveil new targets and opportunities for therapeutic intervention. Our finding that vGPCR angiogenic response is mediated by COX-2 indicates that COX-2 could play a role in KS initiation and progression. This is supported by findings showing that vGPCR activates COX-2 in endothelial cells [41], that KSHV *de novo* infection upregulates COX-2 [31–33,35] and that KSHV-infected malignant cells of human KS lesions express COX-2. Our results showing that COX-2 inhibition block angiogenesis and tumorigenesis induced by the KSHV oncogene vGPCR, constitute a proof of principle for the impact of COX-2 inhibition in KSHV pathogenicity. All these results, together with the expression of COX-2 in KS lesions, contribute to defining COX-2 as a potential target by low-toxicity NSAIDs for the prevention and treatment of KSHV-oncogenesis in HIV infected populations.

## Materials and methods

### Ethics statement

All animal experiments were conducted following the NIH guide for the Care and Use of Laboratory Animals. The animal experiments have been performed under UM IACUC approval number 16–093. The University of Miami has an Animal Welfare Assurance on file with the Office of Laboratory Animal Welfare (OLAW), National Institutes of Health. Additionally, UM is registered with USDA APHIS. The Council on Accreditation of the Association for Assessment and Accreditation of Laboratory Animal Care (AAALAC International) has continued the University of Miami’s full accreditation.

### Cells animals, transfections and chemicals

NIH3T3 and NIH3T3-vGPCR cells were cultured in DMEM + 10% CALF serum. SVEC and SVEC-vGPCR cells were cultured in DMEM + 10% FBS. Transfected cells were maintained in media containing G418 (Sigma, San Louis, Missouri). mECK36 cells were obtained and cultured as described previously [63]. Tetracycline-inducible vGPCR (TET-vGPCR) mECK36 cells were obtained and cultured as previously described [26]. KSHV vGPCR-deleted mutant and its revertant in the Bac16 platform were kindly provided by Drs. Pinghui Feng and J. Jung (Bac16Δ-vGPCR and Bac16Δ-vGPCR-REV). We took advantage of the procedure described in Ashlock *et al*.[47] whereby we swapped Bac36 for Bac16Δ-vGPCR and Bac16Δ-vGPCR-REV. To this end we used KSHV-negative cells (mEC) from frozen populations of KSHV null mECK36 previously obtained after 4 weeks of culturing mECK36 cells without Hygromycin and further selected by weeding and cell sorting and characterized thoroughly for KSHV negativity by PCR for LANA, K1, vIRF-1, ORF23, ORF 36, ORF 74, and K15 [63,70].Cells were transfected using Lipofectamine 3000 Reagent (Thermo, Waltham, Massachusetts) following the manufacturer’s instructions. NS398 (10 μM), PD98059 (20 μM) and SB220025 (10 uM) and SB203580 (10uM) were from Calbiochem (San Diego, California) and Celecoxib (Celebrex¨) was from Pharmacia, Pfizer Inc (New York, New York). Recombinant Human Gro-α/MGSA (25nM) was from Peprotech (Rocky Hill, New Jersey).

### DNA constructs

The plasmid pCOX-2-Luc was provided by Giancarlo V. De Ferrari (Centro de Tecnología e Innovación para el Cáncer (CTI-Cáncer), Department of Biochemistry and Molecular Biology, Faculty of Biological Sciences, Universidad de Concepción, Concepción, Chile) and contains a 1.2 kb murine COX-2 promoter upstream of a luciferase gene as described previously [45]. The plasmid Luc-3’UTR-COX-2 was provided by Dan A. Dixon (Department of Oncological Sciences, Eccles Program in Human Molecular Biology and Genetics, and the Huntsman Cancer Institute, University of Utah, Salt Lake City, Utah) and contains the COX-2 3’UTR region downstream of a luciferase gene as described previously [71]. The plasmid pCEFL-AU5-vGPCR, have been described previously [72] and was a kind gift from J.S. Gutkind laboratory. The expression vectors for the MEKs were previously described [73].

### Luciferase reporter assays

Cells were transfected with different expression plasmids together with 1ug of the indicated reporter plasmid per well in 6-well plates. In all cases, the total amount of plasmid DNA was adjusted with pcDNA3 empty and 0.2ug of pCDNA3-β-galactosidase. Firefly luciferase activity present in cellular lysates was tested using the dual-luciferase reporter system (Promega, Madison, Wisconsin.), and light emission was quantified using a luminometer (Junior Berlthold).

### COX-2 activity assay

Cells were starved overnight (O.N.) in DMEM medium with the addition of the different inhibitors. Cell supernatants were centrifuged at 2,000 x g RT for 5 min. PGE2 levels were measured in those supernatants using the Prostaglandin E2 Express ELISA Kit (Cayman, Ann Arbor, Michigan) following manufacturer’s instructions.

### Western blotting

Protein concentrations in cell lysates were quantified using the DC Protein Assay (Bio-Rad). 20 μg of proteins were mixed with Laemmli buffer, boiled for 5 min, resolved by SDS-PAGE and transferred to PVDF membranes (Bio-Rad Laboratories). Membranes were blocked with 3% BSA for 1 hour and incubated with anti-mouse COX-2 polyclonal antibody (Cayman, Ann Arbor, Michigan), anti-mouse β-actin monoclonal antibody (Sigma, Saint Louis, Missouri), anti-mouse GAPDH monoclonal antibody (Santa Cruz Biotechnology, Inc, Dallas, Texas), anti-mouse ERK1/2 monoclonal antibody (Santa Cruz Biotechnology, Inc, Dallas, Texas) and anti-mouse phospho ERK1/2 monoclonal antibody (Cell Signaling, Danvers, Massachusetts) at 4°C for 16 hours. After 3 TBS/T washes, membranes were incubated with HRP-labeled secondary antibodies (Promega, Madison, Wisconsin) or IRDye Secondary Antibodies (Li-COR, Inc, Lincoln, Nebraska) for 1 hour at room temperature. Protein bands were developed using ECL Plus Detection Reagents (GE Healthcare) or Azure Biosystems C600 Imager.

### Image analysis and quantification

Band intensities corresponding to Western blot detection of protein samples were quantified using the ImageJ software.

### Real-Time quantitative PCR (RT-qPCR)

RNA was isolated with RNeasy Plus Kit (QIAGEN, Valencia, CA). RNA (500 ng) was transcribed into cDNA using Reverse Transcription System (Promega, Madison, Wisconsin) according to the manufacturer’s instructions. RT-qPCR was performed using an ABI Prism 7000 Sequence Detection System (Applied Biosystems) with SybrGreen PCR Master Mix (Quanta Biosciences) using the primers for murine COX-2: 5’-GTGATCGAAGACTACGTGCA-3’ and 5’-TCAGAGGCAATGCGGTTCTG-3, for murine GAPDH: 5’-CAATGACCCCTTCATTGACC-3’ and 5’-GATCTCGCTCCTGGAAGATG-3’, for Firefly luciferase: 5’-CCGCCGTTGTTGTTTTGG-3’ and 5’-ACACAACTCCTCCGCGC-3’, for Renilla Luciferase: 5’-GGAATTATAATGCTTATCTACGTGC-3’ and 5’-CTTGCGAAAAATGAAGACCTTTTAC-3’ and for β galactosidase: 5’-CCACGGAGAATCCGACG-3’ and 5’-GCGAGGCGGTTTTCTCC-3’. In every run, melting curve analysis was performed to verify the specificity of products as well as water and–RT controls. Data were analyzed using the ΔΔCT method as previously described [63]. Target gene expression was normalized to GAPDH by taking the difference between CT values for target genes and GAPDH (ΔCT value). These values were then calibrated to the control sample to give the ΔΔCT value. The fold target gene expression is given by the formula: 2–ΔΔCT.

### Angiogenesis assay

3x10^5^ cells (two inoculation sites per mouse), treated or not with NS398 for 1hour before inoculation, were inoculated intradermally (I.D.) in Balb/C nude mice. Trypan blue (20%) was used to assess cell viability and mark the inoculation site. Mice (5 per group) were sacrificed 5 days later. The area of inoculation was photographed under a dissecting microscope. To assess microvessel density the whole surface area of each section was examined for morphometric analysis following Auerbach’s criteria [74, 75]. To this extent, each photograph slide was projected in a grid corresponding to 1 square mm and the total number of blood microvessels on all grids having vessels was counted. The blood density was defined as the number of vessels per grid (D = total number of vessels / total number of grids counted)[48, 76, 77].

### Tumor growth assay

Mice were inoculated intra-peritoneally (I.P) with Celecoxib 10 mg/kg or vehicle (DMSO). Later the same day they were inoculated subcutaneously (S.C.) with vGPCR transformed NIH3T3 cells (3x10^5^ per mouse). The animals were treated three times a week with Celecoxib (5 mg/kg) or DMSO I.P.[49]. Tumor growth was followed by caliber measurements of volume until the date of sacrifice.

### VEGF production test

Tumor samples were weighted and homogenized in buffer TBS 0.1% BSA with the addition of a protease inhibitor cocktail (Sigma, Saint Louis, Missouri) in a volume proportional to their weight. Cell supernatants were assayed after centrifugation at 2,000 x g RT for 5 min. ELISA with anti-mouse-VEGF (R&D Systems, Minneapolis, Minnesota) was performed following the manufacturer’s instructions. VEGF levels from tumor samples were normalized based on lysate protein concentration.

### Immunohistochemistry of angiogenic markers

Frozen sections of the tumors were fixed in acetone and an immunoperoxidase staining was performed by standard immunohistochemistry methods. Briefly: after blocking, samples were incubated ON with anti-CD31 (Pharmingen, San Diego, CA), anti-α-SMA (Sigma, San Louis, Missouri) or isotype-matched control antibodies as indicated. After 30 min of incubation with goat anti rat-IgG (Pharmingen, San Diego, CA) or anti mouse-IgG kit (Vector, Burlingame, California), for CD-31 or α-SMA respectively. Sections were developed using *Elite*-Vectastain ABC-peroxidase (Vector, Burlingame, CA) for 30 min and DAB substrate (Vector, Burlingame, CA). Slides were counterstained with hematoxylin and mounted. Pictures were taken using an Olympus microscope equipped with a digital camera.

### Immunofluorescence staining

Immunofluorescence assay (IFA) was performed as previously described [63]. Briefly, cells were fixed in 4% paraformaldehyde for 10 min and washed with PBS. Cells were permeabilized in 0.2% Triton-X/PBS for 20 min at 4°C. After blocking with 3% of BSA in PBS and 0.1% Tween 20 for 60 min, samples were incubated with primary antibodies overnight at 4C. After PBS washing, samples were incubated with fluorescent secondary antibodies for 1 hour (Molecular Probes), washed and mounted with ProLong Gold antifade reagent with DAPI (Molecular Probes). Images were taken using a Zeiss ApoTome Axiovert 200M microscope.

### COX-2 and KSHV LANA double Immunohistochemistry

Immunohistochemical staining was performed on formalin-fixed paraffin-embedded biopsy specimens of human KS lesions (1 skin, 3 lymph nodes, and 1 lung) from the tissue repository of the Department of Pathology and Laboratory Medicine at the New York Presbiterian Hospital-Weill Cornell Medical Center. Immunohistochemical staining was performed on a TechMate 500 automated immunostainer (Ventana Medical Systems, Tucson AZ). For the COX-2 staining, sections were treated in a DAKO Antigen Retrieval Solution (DakoCytomation, Carpinteria, CA) and COX-2 was detected using an anti-human COX-2 antibody (Zymed Laboratories, San Francisco, CA) followed by an HRP-labelled secondary antibody detection system and DAB chromogen (DakoCytomation). For the second round of analysis by IHC, KS tissue sections were again retrieved with a DAKO Antigen Retrieval Solution (DakoCytomation) and KSHV-LANA was detected using a rat monoclonal to LANA-1 ORF 73 (Advanced Biotechnologies, MD), a secondary anti-rat biotinylated antibody (BD Pharmingen) and developed using an ABC Alkaline-Phosphatase complex (Ventana) and BT Red Reagent substrate (Ventana). Slides were counterstained with hematoxylin and mounted. Pictures were taken using an Olympus microscope equipped with a camera.

### Statistical analysis

Statistical significance of the data was determined using a two-tailed Student’s t-test and 2way ANOVA for multiple comparisons. A p-value lower than 0.05 was considered significant. Statistical analysis was performed using GraphPad Prism 7. All the experiments were repeated at least three times for consistency. All values were expressed as means ± standard deviation.

## Supporting information

S1 Fig Fold-changes in mRNA expression determined by RT-qPCR in mECK16 derived cells (Δ-vGPCR or revertant virus). LANA (A) or vFLIP (B) mRNA expression levels were measured in triplicate and are presented as means ± SD. (*P<0.05).
(TIF)

S2 Fig Immunoperoxidase staining for COX-2 or Isotype Control were performed on vGPCR-induced tumor sections. (TIF)
