# Supplementary figures and images for: KSHV G-protein coupled receptor vGPCR oncogenic signaling upregulation of Cyclooxygenase-2 expression mediates angiogenesis and tumorigenesis in Kaposi’s sarcoma
Source: PLoS Pathog. 2020 Oct 15;16(10):e1009006. doi: 10.1371/journal.ppat.1009006 (PMC7591070; doi:10.1371/journal.ppat.1009006)

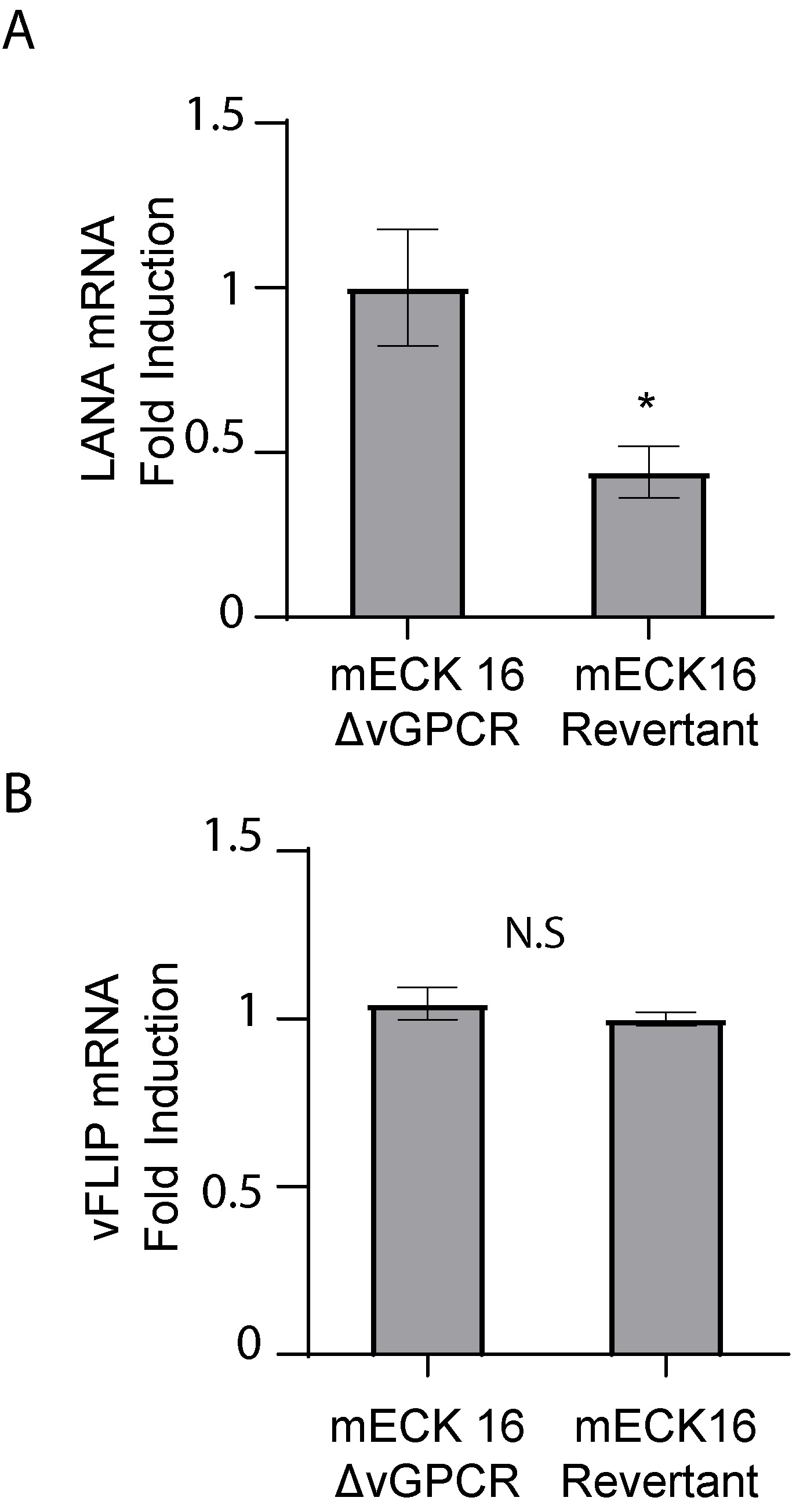

Supplement: S1 Fig — LANA (A) or vFLIP (B) mRNA expression levels were measured in triplicate and are presented as means ± SD. (*P<0.05). (TIF) [file ppat.1009006.s001.tif]

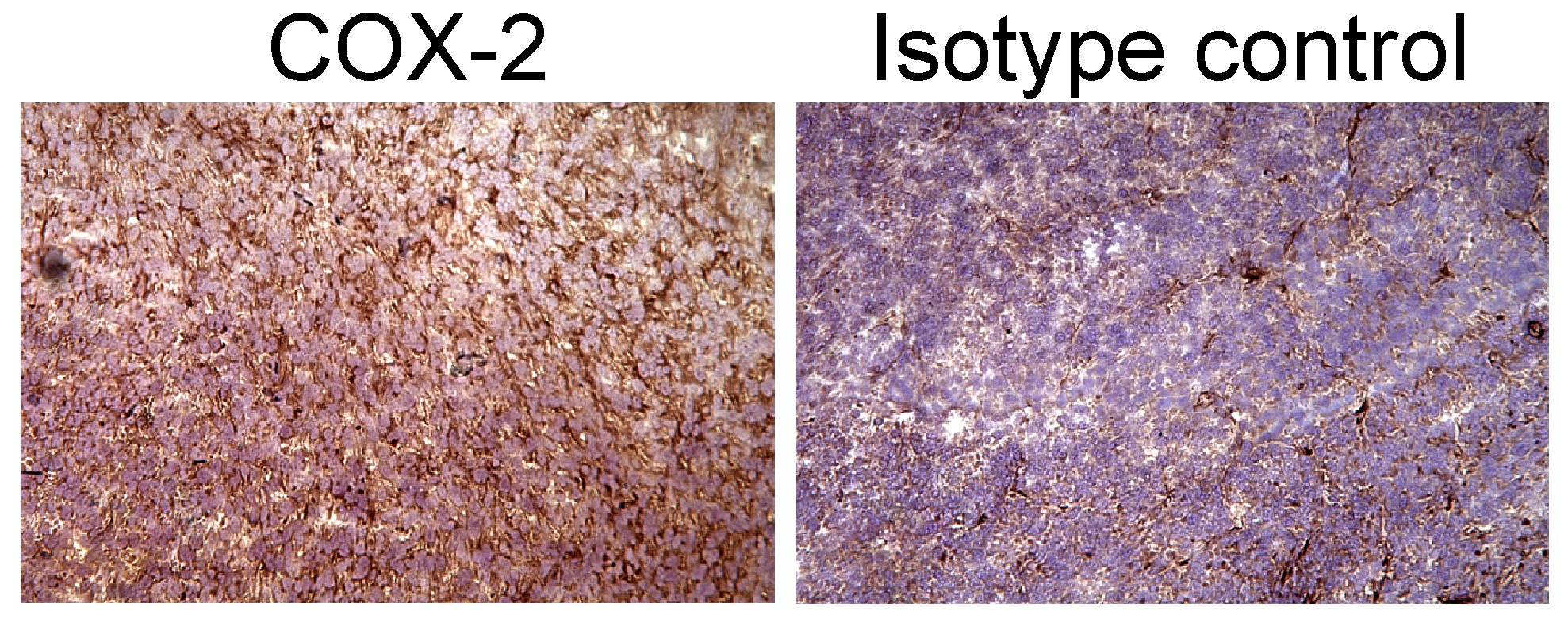

Supplement: S2 Fig — (TIF) [file ppat.1009006.s002.tif]
